# The Impact and Challenges of the Implementation of 5S Methodology in Healthcare Settings: A Systematic Review

**DOI:** 10.7759/cureus.64634

**Published:** 2024-07-16

**Authors:** Bhavesh Kanabar, Kiran G Piparva, Dhruvin Pandya, Rajvi B Kanabar

**Affiliations:** 1 Preventive and Social Medicine, Pandit Deendayal Upadhyay (PDU) Government Medical College, Rajkot, IND; 2 Pharamacology, All India Institutes of Medical Sciences (AIIMS), Rajkot, IND; 3 Medicine, Pandit Deendayal Upadhyay (PDU) Government Medical College, Rajkot, IND; 4 Family Medicine, Rajkot Municipal Corporation, Rajkot, IND

**Keywords:** implementation, quality improvement, lean management, healthcare settings, 5s methodology

## Abstract

The 5S methodology, rooted in lean manufacturing principles, has been adopted in healthcare settings to improve organization, efficiency, and quality. This systematic review aimed to synthesize the literature on the implementation of the 5S methodology in healthcare, its impact, and factors influencing successful implementation.

A comprehensive literature search was conducted in PubMed, Google Scholar, and Cochrane databases for original studies on the implementation of the 5S methodology in healthcare settings. Studies were screened based on inclusion and exclusion criteria, and data were extracted on study characteristics, implementation details, outcomes, and key findings.

Six studies met the inclusion criteria, spanning various healthcare settings, including hospitals, clinics, and laboratories. The studies reported positive outcomes associated with implementing the 5S methodology, such as improved workplace organization and cleanliness, increased utilization of healthcare services, enhanced staff satisfaction and motivation, and reduced waste and non-conformities. Critical success factors included commitment from top management, staff involvement, continuous monitoring, and adequate training. However, challenges were noted, including limited resources, small sample sizes, and difficulty assessing long-term sustainability.

The implementation of the 5S methodology in healthcare settings can lead to improved organization, efficiency, and quality. However, successful implementation requires addressing critical factors such as leadership commitment, staff engagement, continuous monitoring, and training. Future research should focus on larger-scale implementation studies, long-term assessments, quantitative and qualitative evaluations, and cost-effectiveness analyses to strengthen the evidence base and inform best practices.

## Introduction and background

**Figure 1:**
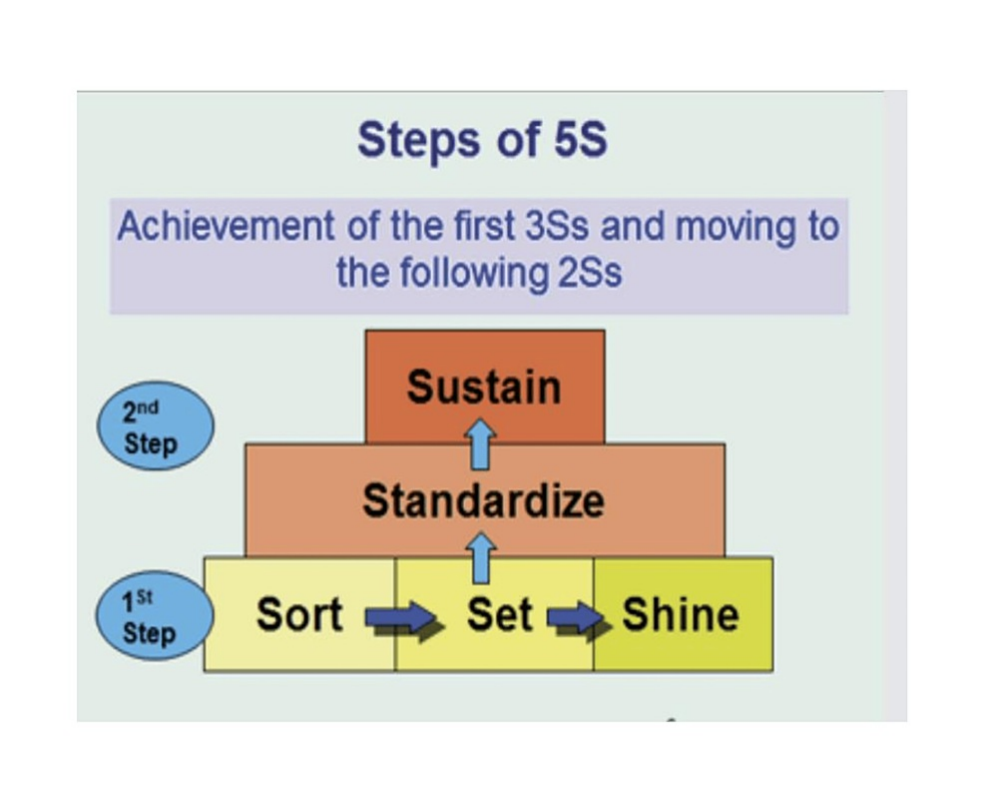
The 5S methodology stands for Sort, Set in Order, Shine, Standardize, and Sustain. Figure 1 illustrates the steps for achieving the 5S components: Sort, Set in Order, Shine, Standardize, and Sustain.

Healthcare systems worldwide face challenges related to quality, safety, and efficiency, driven by increasing demands, limited resources, and the need for continuous improvement [1,2]. The 5S methodology, originating from the lean manufacturing principles of the Toyota Production System, has emerged as a promising approach to address these challenges in healthcare settings. The 5S methodology focuses on creating and maintaining an organized, clean, and efficient workspace by following five principles: Sort (Seiri), Set in Order (Seiton), Shine (Seiso), Standardize (Seiketsu), and Sustain (Shitsuke) [3,4]. Sort (Seiri) involves identifying and removing unnecessary items from the workspace, while Set in Order (Seiton) ensures that necessary items are arranged in a logical and easily accessible manner. Shine (Seiso) emphasizes maintaining cleanliness and orderliness in the workspace, while Standardize (Seiketsu) establishes consistent practices and procedures. Finally, Sustain (Shitsuke) promotes the ongoing application and continuous improvement of the 5S principles [3,4] (Figure 1).

The 5S management method, regarded as the cornerstone of lean healthcare strategies, aims to enhance value-added processes by eliminating non-value-adding elements [5]. It has been adapted for use in healthcare settings as a systematic approach to organizing and standardizing work environments in line with lean healthcare principles [6]. Recognized for its cost-effectiveness and simplicity, it serves as a fundamental starting point for enhancing healthcare services [6-10].

The implementation of the 5S methodology in healthcare settings has been reported to yield various benefits, including improved organization, efficiency, and safety, reduced waste and costs, enhanced staff motivation and satisfaction, and improved quality of care [11,12]. However, successful implementation requires commitment from leadership, staff involvement, and continuous efforts [12,13]. The impact of the application of the 5S management method in the healthcare sector has been documented in the United States [14-17], India [18], Jordan [19], and Sri Lanka [20]. Despite these findings, there is limited understanding regarding the detailed implementation of the 5S management method and its direct impact on the quality of healthcare services. This systematic review aims to synthesize the available literature on the implementation of the 5S methodology in healthcare settings, its impact on various outcomes, and the factors influencing its successful implementation.

## Review

Methods

We followed the Preferred Reporting Items for Systematic Reviews and Meta-Analyses (PRISMA) guidelines. A thorough literature search for studies on the implementation of the 5S methodology in healthcare settings was independently conducted by two reviewers (BK and KP) in PubMed, Google Scholar, and Cochrane databases, covering publications up to March 2024.

The database search was conducted using the keywords "5S methodology," "Healthcare," "Hospitals," and "Implementation," following the specified search strategy.

Search Strategy

The search strategy was as follows: "5S"[All Fields] AND "implementation"[All Fields] AND ("delivery of health care"[MeSH Terms] OR ("delivery"[All Fields] AND "health"[All Fields] AND "care"[All Fields]) OR "delivery of health care"[All Fields] OR ("health"[All Fields] AND "care"[All Fields]) OR "health care"[All Fields]) AND ("setting"[All Fields] OR "settings"[All Fields] OR "settings"[All Fields]).

Study Selection

The original research articles published in peer-reviewed journals or conference proceedings and accessible as full-text studies in any language were included. Studies other than original articles (review articles, editorials, and opinion pieces) exploring the implementation of the 5S methodology in settings other than healthcare were excluded.

**Figure 2:**
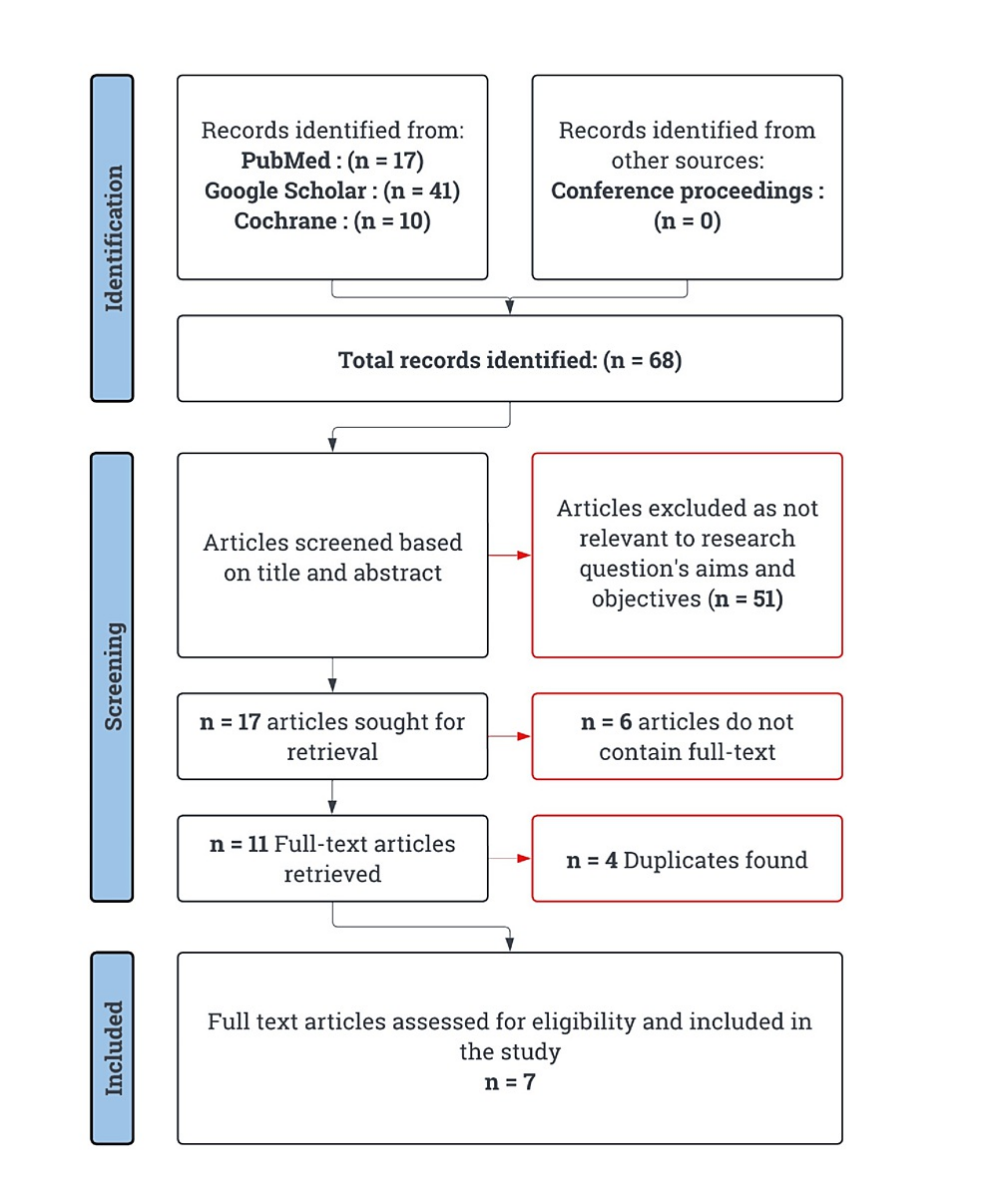
PRISMA flowchart for study selection PRISMA: Preferred Reporting Items for Systematic Reviews and Meta-Analyses Reference: [21]

The electronic literature search identified 17 original research articles from PubMed, 41 from Google Scholar, and 10 from the Cochrane database. Seven studies met the inclusion criteria and were included in the final analysis (Figure 2).

Data Extraction and Quality Assessment

Two independent reviewers (RK, KP) extracted data from the eligible studies, including study characteristics (authors, year, study design, setting, sample size), intervention details, outcome measures, and key findings. Any discrepancies were resolved through discussion or consultation with a third reviewer (BK). The methodological quality of the included studies was assessed by using quality assessment tools, the Joanna Briggs Institute (JBI), and Critical Appraisal Checklists according to the study design [22,23]. 

Results

After screening the titles, abstracts, and full texts, six studies met the inclusion criteria. Upon careful re-reading and thorough analysis, the author noted variations in study designs (including study settings, hypotheses, and implementation approaches), study participants (covering staff composition and methods of monitoring and evaluation related to the implementation of the 5S), and study impacts (encompassing outcomes, research gaps, limitations, and implications).

*Study Design* 

The included studies were conducted in various healthcare settings, including healthcare centers [13,11], hospitals [24-26], and clinical laboratories [27,12]. The study designs included cross-sectional studies [13], observational studies [24,25,27], qualitative studies [11], and educational interventions [26]. As per the study setup, implementation of 5S has objectives of impact on the utilization of healthcare services [13], impact on employees' satisfaction [27], impact on the work environment [12], impact of introducing the 5S-KAIZEN into the public health curriculum [26], and impact on the functionality of sector [24] (Table 1).

**Table 1:** Study design, setting, and objectives Table 1 provides detailed information about the authors, country of study, type of healthcare setting, study design methodology, and primary hypotheses and objectives References: [13,11,25,12,26,27,24]

Study	Region	Study setting	Study design	Hypotheses tested	Study objectives
Pandya et al. [13]	India	Urban health centers (multicentric)	Cross-sectional observational study	Impact of 5S implementation on healthcare service utilization	To assess the status of 5S implementation and its impact on healthcare service utilization
Kanamori et al. [11]	Senegal	Health center (multicentric)	Qualitative study to assess the progress of 5S	5S components and impact on service utilization	Implementation of 5S components, an impact on service utilization
Chinda et al. [25]	Thailand	Hospitals (multicentric)	Survey-type observational study	5S improves quality and motivation in disorderly settings	To assess how 5S creates changes in the workplace, processes, and outcomes
Singh et al. [12]	India	Clinical Biochemistry Laboratory at Government Institute (single center)	Interventional study	5S implementation is not affected by hospital size	To investigate 5S implementation in hospitals
El-Sherbiny et al. [26]	Egypt	Medical school (single center)	Educational intervention study	5S implementation improved the staff perception effectiveness and feasibility positively	Perceptions, feasibility, and effectiveness of 5S implementation
Dogan et al. [27]	Turkey	Clinical laboratory (single center)	Observational study	Testing the impact of 5S implementation in the medical curriculum	Impact of 5S on employees' satisfaction considering the areas such as facilitating the job, job satisfaction, setting up a safe environment, and the effect of participation in management
Alves et al. [24]	Brazil	Medical and surgical clinic unit in a teaching hospital (single center)	Observational study	5S enhances and sustains sectoral orderliness and organization	To implement 5S and evaluate effects on the organization and rationalization of the work environment

Methods of 5S Implementation in Healthcare Setups

The 5S approach involves training and workshops to prepare centers before implementation. Most studies followed a phased, step-by-step implementation of 5S within their respective setups, namely, Phase 1: initial planning and training for 5S implementation; Phase 2: practice of 5S implementation; and Phase 3: evaluation of 5S impact in their respective objectives.

The study done by Kanamori et al. implemented 5S in three phases: a one-day training workshop for 62 staff and then hands-on implementation at nine locations which were then preceded by two assessment meetings involving staff, government officials, and experts [11].

The study conducted by Pandya et al. involved a 5S campaign, with Phase 1 seeing the municipal corporation implementing the 5S approach across all 18 urban health centers in Rajkot city, Phase 2 focusing on practicing 5S at all centers, and Phase 3 concentrating on assessing 5S using a structured audit checklist [13].

The study conducted by El-Sherbiny et al. was carried out in six phases: identifying the problem of medical education's focus on clinical examination without workplace management; conducting professional and market needs assessments; setting the course goals and objectives; formulating educational strategies and structuring the 5S-KAIZEN course; successfully implementing the 5S approach; and, finally, conducting evaluation and gathering feedback [26].

The study conducted by Singh et al. involved an eight-stage approach: stages 1-5 focused on planning and preparation, including staff training; stages 6 and 7 on the implementation and practice of 5S; and stage 8 on feedback and review of the 5S process [12].

Alves et al. conducted a study to implement and evaluate 5S in the organization and functionality of surgical and medical units, based on the framework developed by Melleiro (2005) comprising four phases: explanatory, normative, strategic, and tactical-operational [24].

The majority of study participants were hospital staff at healthcare centers [11,13,24-26] and staff at clinical laboratories [27,12] (Table 2).

**Table 2:** Study objectives and methodology (intervention, main findings, and outcomes) Table 2 outlines the main objectives of the studies and provides detailed information about the methodology, including the intervention, study assessment tools, main findings, and study outcomes References: [13,11,25,12,26,27,24]

Study	Participants involved in the implementation	Sample size	Intervention	Assessment tool	Main findings
Pandya et al. [13]	Individuals utilizing public healthcare services in urban areas	18 urban health centers	5S campaign implementation in urban health centers	Structured audit checklist recommended by State Quality Assurance Cell and Observation of performance indicators for staff	Significant improvement in score of all components of 5S and utilization of major health services
Kanamori et al. [11]	Health center staff members	21 health centers	5S pilot intervention in a health facility	Qualitative interviews	5S improved the work environment, attitude and behavior of staff, and quality of services
Chinda et al. [25]	Hospital staff	150 hospitals	5S implementation in hospitals	Questionnaire survey, factor analysis	5S contributes to organization, efficiency, and no variation by hospital size
Singh et al. [12]	Healthcare workers (faculty members, technicians, data entry operators, and a storekeeper in a clinical biochemistry laboratory)	15 participants	5S implementation in a clinical biochemistry laboratory	Feedback questionnaire	5S led to an organized, safe workplace and positive staff attitudes
El-Sherbiny et al. [26]	Fourth-year medical students	100 participants	5S-KAIZEN curriculum integration for medical students	Evaluation of written reports and performance checklist	5S-KAIZEN prepares graduates for quality improvement
Dogan et al. [27]	Laboratory staff	63 laboratory staff	5S implementation in a clinical laboratory	Questionnaire	5S improved employee satisfaction and reduced non-conformities
Alves et al. [24]	Hospital unit staff, patients, and companions	Not mentioned	5S implementation in a medical and surgical clinic unit	Survey by Situational Strategic Planning (PES)	5S led to an organized, functional sector in the hospital

Factors Influencing Successful Implementation

The reviewed studies identified several factors that influence the successful implementation of the 5S methodology in healthcare settings.

Commitment from top management: Several authors emphasized the importance of commitment from top management for successful implementation [12,13]. Pandya et al. stated that the 5S practice can be sustained with sincere and continuous efforts highlighting the role of leadership commitment [13].

Training and education: Providing adequate training and education to healthcare staff was identified as a critical factor for successful implementation. Kanamori et al. highlighted the need for promoting knowledge about 5S among healthcare providers [11]. El-Sherbiny et al. described integrating 5S-KAIZEN concepts into the medical curriculum to prepare students for continuous quality improvement [26].

Staff involvement and participation: Staff involvement and participation were highlighted as crucial factors for the successful implementation of the 5S methodology [12]. Singh et al. attributed the success of their intervention to commitment from top management and staff involvement [12]. Dogan et al. recommended the use of 5S methods to encourage employee participation in the management process [27].

Continuous efforts and monitoring: Maintaining the gains achieved through the 5S implementation requires continuous efforts and monitoring. Pandya et al. emphasized that the 5S practice can be sustained with sincere and continuous efforts [13]. Singh et al. recommended conducting feedback and follow-up activities for sustained use of the methodology [12].

Outcomes and Impact of 5S Implementation in Healthcare Setting

​​​​​The evaluation of the implementation of the 5S methodology in healthcare settings utilized a variety of methods, including quantitative data collection methods such as questionnaires, checklists, and surveys, alongside qualitative methods such as staff interviews (Table 2).

The reviewed studies reported various positive outcomes and impacts associated with the implementation of the 5S methodology in healthcare settings (Table 3).

**Table 3:** Study outcome and impact: intervention effects, study limitation, research gaps, and policy recommendations Table 3 summarizes the overall impact of the studies, including intervention effects, research gaps, limitations, and policy recommendations References: [13,11,25,12,26,27,24]

Study	Intervention effects	Limitations	Research gaps	Policy recommendations
Pandya et al. [13]	Significant improvement in utilization in major healthcare services	Possibility of subjectivity in scoring, assessors differed	Not mentioned	Adopt 5S strategies and sustain with continuous efforts
Kanamori et al. [11]	Improved work environment, quality of healthcare services, and staff motivation	Small sample size	Larger-scale studies are required to validate the findings and to identify the cost-effectiveness approach for integration	Further quantitative and qualitative research based on larger-scale intervention and cost-effectiveness
Chinda et al. [25]	Implementing 5S enhances safety standards	Not mentioned	Not mentioned	5S standard is considered one of the important safety standards implemented in hospitals
Singh et al. [12]	Improved the understanding of the key concepts of 5S among staff members and built positive attitudes	Single center, small sample, short study period, novelty effect	A longer study period would help observe if the last "S (Sustain)" of 5S is holding good or not	Commitment from management, staff involvement, follow-up activities
El-Sherbiny et al. [26]	Change in students' knowledge, attitudes, and behaviors	Limited time, limited support, assessing long-term impact	Limited time, support, assessing long-term impact	Training programs for students and nursing in large group
Dogan et al. [27]	Improved satisfaction and reduced non-conformities	Small sample, no control group, generalizability	Long-term sustainability	Use 5S for a safe environment, job facilitation, staff participation
Alves et al. [24]	Organized, functional sector positively impacting the team's work of health	Not mentioned	Many employees were resistant to the proposed changes	Consider 5S implementation in healthcare facilities

Improved workplace organization and cleanliness: Several studies highlighted the positive impact of the 5S methodology on workplace organization and cleanliness. Pandya et al. reported significant improvements in all five components of 5S, with the highest improvement in Sorting and Setting in Order [13]. Alves et al. noted that the implementation of the 5S tool led to a more organized and functional sector by removing damaged and disused materials and arranging equipment in designated places [24].

Increased utilization of healthcare services: The implementation of the 5S methodology was also associated with increased utilization of healthcare services. Pandya et al. found a significant improvement in the utilization of major healthcare services such as outpatient, laboratory services, immunization, and family planning services after implementing the 5S Campaign [13].

Improved staff satisfaction and motivation: Several studies reported positive impacts on staff satisfaction and motivation following the implementation of the 5S methodology. Kanamori et al. found that the 5S management method was perceived to have improved staff motivation in a resource-poor healthcare facility in Senegal [11]. Dogan et al. reported a statistically significant improvement in employee satisfaction after implementing 5S methods in a clinical laboratory in Turkey [27].

Reduced waste and non-conformities: The 5S methodology was associated with reduced waste and non-conformities in healthcare settings. Dogan et al. reported a significant reduction of 69.7% in the non-conformity score in the laboratory after implementing 5S methods [27].

Challenges and limitations: While the reviewed studies reported positive outcomes, they also acknowledged several challenges and limitations associated with implementing the 5S methodology in healthcare settings [11].

Limited allocated time and resources: Some studies reported limited allocated time and resources as challenges. El-Sherbiny et al. mentioned limited allocated time for teaching the course and limited support and cooperation from healthcare personnel during practical application [26].

Small sample size and limited generalizability: Several studies had small sample sizes which limits the generalizability of their findings. Dogan et al. acknowledged the "small sample size" and "lack of a control group or comparison" as limitations [11,27].

Difficulty in assessing long-term sustainability: While the reviewed studies reported positive outcomes, assessing the long-term sustainability of the 5S implementation was a challenge. Dogan et al. noted the lack of information on long-term sustainability as a limitation [27].

Implications for Practice and Policy

The findings of this systematic review have several implications for practice and policy in healthcare settings (Table 3).

Adoption of the 5S methodology: Healthcare organizations should consider adopting the 5S methodology as a lean management approach to improve workplace organization, cleanliness, safety, staff satisfaction, and overall efficiency. The potential benefits of the 5S methodology, as demonstrated in the reviewed studies, warrant its implementation in various healthcare settings, such as hospitals, clinics, and laboratories [11,28].

Leadership commitment and staff engagement: Successful implementation of the 5S methodology requires commitment from top management and active engagement of healthcare staff at all levels. Healthcare organizations should develop strategies to promote leadership commitment, staff participation, and continuous monitoring of the 5S implementation [12,28].

Training and education: Adequate training and education on the 5S principles and implementation methods should be provided to healthcare professionals and support staff. Integrating 5S concepts into medical and nursing curricula can prepare future healthcare professionals for continuous quality improvement initiatives [26].

Resource allocation: Healthcare organizations should allocate appropriate resources, including time, personnel, and funding, for the effective implementation and sustained practice of the 5S methodology. Policymakers and healthcare administrators should consider the potential benefits of the 5S methodology when allocating resources for quality improvement initiatives [11,29].

Continuous monitoring and evaluation: Continuous monitoring and evaluation of the 5S implementation are crucial for sustaining the gains and identifying areas for improvement. Healthcare organizations should develop standardized processes for monitoring and evaluating the impact of the 5S methodology on various outcomes, such as staff satisfaction, patient safety, and operational efficiency [11,28].

Collaboration and knowledge sharing: Healthcare organizations should foster collaboration and knowledge sharing among institutions that have implemented the 5S methodology. Best practices, lessons learned, and effective strategies can be shared to support successful implementation in other healthcare settings [11,25].

Policy support and incentives: Policymakers and healthcare regulatory bodies should consider providing incentives and support for healthcare organizations that adopt lean management approaches, such as the 5S methodology, to promote continuous quality improvement and efficient resource utilization [11,30].

Research Gaps

The reviewed studies identified several research gaps and highlighted the need for future research in the following areas:

​​Small-scale studies: Kanamori et al. and Dogan et al. emphasized the need for larger-scale implementation studies to validate findings and assess the cost-effectiveness of the 5S methodology in healthcare settings [11,27].

Cross-sectional studies: The included studies were assessed at one point in time, i.e., cross-sectional studies. Future research should focus on long-term follow-up studies to assess the sustainability of the 5S implementation and its impact over an extended period [27].

Discussion

The findings of this systematic review suggest that the implementation of the 5S methodology in healthcare settings can lead to positive outcomes, such as improved organization, cleanliness, and safety in the workplace, increased staff satisfaction and motivation, reduced wastage, and enhanced utilization of healthcare services. These findings are consistent with the principles of the 5S methodology, which aims to create an organized, efficient, and safe work environment [4]. Several studies reported improvements in the utilization of healthcare services after implementing the 5S methodology [13]. This observation aligns with the notion that an organized and efficient work environment can facilitate better service delivery and patient care [4].

The positive impact on staff satisfaction and motivation was reported in several studies [11,27]. It is noteworthy, as employee engagement and job satisfaction are crucial factors in healthcare settings, directly influencing the quality of care and patient outcomes [31].

The reduction in waste and non-conformities observed after implementing the 5S methodology [4,27] aligns with the lean principles of eliminating waste and improving efficiency, which can contribute to cost savings and better resource utilization in healthcare organizations [31].

The successful implementation of the 5S methodology in healthcare settings hinges on several critical factors, including commitment from top management, staff involvement and participation, continuous efforts and monitoring, and adequate training and education [11-13,26]. These factors are consistent with the principles of organizational change management and the importance of leadership, employee engagement, and capacity building in driving sustainable improvements.

Despite the reported positive outcomes, the reviewed studies acknowledged several challenges and limitations, such as limited allocated time and resources, small sample sizes, limited generalizability, and difficulty in assessing long-term sustainability [11,26,27]. These limitations highlight the need for larger-scale implementation studies, long-term follow-up assessments, quantitative and qualitative evaluations, comparative studies across different healthcare settings, and cost-effectiveness analyses.

Limitations of the review

While this systematic review aimed to provide a comprehensive synthesis of the available literature on the implementation of the 5S methodology in healthcare settings, it is important to acknowledge some limitations.

Publication Bias

Like most systematic reviews, this review may be subject to publication bias, where studies with positive or significant findings are more likely to be published than those with null or negative results. This bias could lead to an overestimation of the positive effects associated with the implementation of the 5S methodology.

Quality Assessment Challenges

The assessment of methodological quality in the included studies was challenging due to the diverse study designs and the lack of standardized quality assessment tools specific to the implementation of quality improvement interventions in healthcare settings.

Heterogeneity of Study Designs and Outcomes

The included studies employed a variety of study designs, ranging from cross-sectional studies to qualitative investigations and educational interventions. Additionally, the outcomes measured and reported varied across studies, making it difficult to perform meta-analyses or quantitative syntheses.

Limited Generalizability

Some of the included studies were conducted in specific healthcare settings or geographic regions, limiting the generalizability of their findings to other contexts. The transferability of the results to different healthcare systems, cultures, and resource settings may be limited.

Lack of Cost-Effectiveness Data

Most of the included studies did not report data on the cost-effectiveness of implementing the 5S methodology in healthcare settings. This limitation hinders a comprehensive understanding of the economic implications and potential cost savings associated with the implementation of the 5S methodology.

Reliance on Secondary Data Sources

This systematic review relied on published literature and secondary data sources. The accuracy and completeness of the information reported in the included studies could not be independently verified, potentially introducing biases or inaccuracies.

Despite these limitations, this systematic review provides a valuable synthesis of the current literature on the implementation of the 5S methodology in healthcare settings. The findings highlight the potential benefits, critical success factors, and research gaps, contributing to the understanding of this quality improvement approach and informing future research and practice.

Future research directions

Based on the research gaps and limitations identified in this systematic review, the following future research directions are recommended.

Large-Scale Implementation Studies

To confirm the findings and evaluate the generalizability of the results, large-scale studies with larger sample sizes are necessary. These studies should implement the 5S methodology across diverse healthcare settings, including hospitals, clinics, laboratories, and long-term care facilities [13]. 

Long-Term Follow-Up Assessments

Long-term follow-up studies are required to evaluate the sustainability of 5S implementation and its long-term impact on various outcomes, such as staff satisfaction, patient safety, quality of care, and operational efficiency, over an extended period [12,13].

Comparative Studies

Comparative studies examining the implementation of the 5S methodology across different healthcare settings, regions, and countries could provide valuable insights into the generalizability and context-specific factors influencing its success [11,12].

Cost-Effectiveness Analyses

Future research should include cost-effectiveness analyses to evaluate the economic benefits of implementing the 5S methodology in healthcare settings, particularly in resource-constrained environments [11].

Integration With Other Lean Methodologies

Explore the integration of the 5S methodology with other lean management tools and techniques, such as value stream mapping, process mapping, and root cause analysis. This combination can offer a comprehensive approach to continuous quality improvement and process optimization in healthcare settings.

Exploring Enablers and Barriers

Investigate the enablers and barriers to successful implementation of the 5S methodology in healthcare settings. These studies should focus on identifying organizational, cultural, and individual factors that facilitate or hinder the adoption and sustained use of the 5S principles, informing strategies for effective implementation.

Development of Implementation Frameworks and Guidelines

Based on the findings from future research, develop implementation frameworks and guidelines tailored to different healthcare settings. These frameworks should provide practical guidance on the step-by-step implementation process, training requirements, monitoring and evaluation strategies, and best practices for sustaining the 5S methodology.

By addressing these future research directions, the body of evidence on the implementation of the 5S methodology in healthcare settings can be strengthened, informing evidence-based practices and policies to enhance quality, safety, and efficiency in healthcare delivery.

## Conclusions

The 5S methodology can lead to positive outcomes in the healthcare setting such as improved workplace organization, cleanliness, safety, staff satisfaction, reduced waste, and enhanced utilization of healthcare services. However, the review also highlights key prerequisites for successful implementation, including top management commitment, staff involvement, ongoing efforts, adequate training, and monitoring. While the reviewed studies reported promising results, several limitations and research gaps were identified, highlighting the need for larger-scale implementation studies, long-term follow-up assessments, quantitative and qualitative evaluations, comparative studies across different healthcare settings, and cost-effectiveness analyses. Healthcare organizations should consider adopting the 5S methodology as a lean management approach to improve quality, safety, and efficiency. Policymakers and healthcare administrators should allocate appropriate resources, provide incentives, and support the implementation of the 5S methodology to promote continuous quality improvement and efficient resource utilization in healthcare settings.
